# Effect of tart cherry juice on recovery and next day performance in well-trained Water Polo players

**DOI:** 10.1186/s12970-016-0151-x

**Published:** 2016-11-14

**Authors:** Rachel McCormick, Peter Peeling, Martyn Binnie, Brian Dawson, Marc Sim

**Affiliations:** 1 School of Sport Science, Exercise and Health, The University of Western Australia, Crawley, Western Australia Australia; 2 Western Australian Institute of Sport, Mt Claremont, Western Australia Australia; 3 School of Health Sciences and Psychology, Federation University, Mt Helen, Victoria Australia

**Keywords:** Supplement, Inflammation, Oxidative stress, Team-sports

## Abstract

**Background:**

Tart Montmorency cherries contain high concentrations of phytochemicals and anthocyanins, which have recently been linked to improved athletic recovery and subsequent performance. To date however, previous work reporting promising results has focused on land-based endurance sports, with any potential benefits to team sports remaining unknown. As such, this investigation set-out to examine the effect of supplemental tart cherry juice (CJ) on recovery and next day athletic performance in highly-trained water-based team sport athletes over seven days.

**Methods:**

In a randomised, double-blind, repeated measures, crossover design, nine male Water Polo athletes were supplemented with CJ or a placebo equivalent (PLA) for six consecutive days. Prior to, and at the completion of the supplementation period, water-based performance testing was conducted. On day 6, participants also undertook a fatiguing simulated team game activity. Venous blood samples were collected (Pre-exercise: day 1, 6 and 7; Post-exercise: day 6) to investigate markers of inflammation [Interleukin-6 (IL-6); C-reactive protein (CRP)] and oxidative stress [Uric Acid (UA); F2-Isoprostane (F2-IsoP)]. A daily diary was also completed (total quality of recovery, delayed onset muscle soreness) as a measure of perceptual recovery.

**Results:**

In both conditions, day 6 post-exercise IL-6 was significantly higher than pre-exercise and day 7 (*p* < 0.05); CRP was greater on day 7 as compared to day 6 pre- and post-exercise (*p* < 0.05); F2-IsoP was significantly lower on day 7 as compared to day 1 and day 6 (*p* < 0.05); UA remained unchanged (*p* > 0.05). No differences were found for any performance or recovery measures.

**Conclusions:**

The lack of difference observed in the blood markers between groups may reflect the intermittent, non-weight bearing demands of Water Polo, with such activity possibly unable to create a substantial inflammatory response or oxidative stress (over 7 days) to impede performance; thereby negating any potential beneficial effects associated with CJ supplementation.

**Trial registration:**

This trial was registered with the Australian and New Zealand Clinical Trials Registry (ANZCTR). Registration number: ACTRN12616001080415. Date registered: 11/08/2016, retrospectively registered.

## Background

Tart Montmorency cherries have recently gained repute as a useful supplement for athletes due to their high concentration of phytochemicals and anthocyanins, which have been linked to numerous health benefits. These include high anti-inflammatory and anti-oxidant capacities, enhanced sleep, improved recovery and a reduction in post-exercise muscle damage and soreness [1–3]. Such findings have recently led to tart cherry juice (CJ) being included as a ‘Group B’ supplement (warrants further research/provided to athletes within research or clinical monitoring situations) on the Australian Institutue of Sport Supplement Classification System. To date however, all exercise-based studies have investigated the effects of CJ supplementation on recovery from maximal strength or endurance (>60 min duration) exercise, demonstrating an attenuation of markers related to both inflammation and oxidative stress [2, 4, 5]. As such, any response linked to accelerated recovery would appear beneficial when considering the large training load experienced by high performance athletes.

Despite the aforementioned benefits of tart CJ supplementation, its effect on performance, inflammation and oxidative stress in water-based team sport athletes remains unknown. Specifically, the non-weight bearing intermittent nature of Water Polo may influence the typical post-exercise inflammatory and oxidative stress response (compared to endurance exercise), thus having implications for the type of recovery strategies adopted by such populations. Therefore, the aim of this investigation was to assess the effect of supplemental tart CJ on athletic recovery and next day performance in highly-trained Water Polo players. It was hypothesised that tart CJ supplementation (in comparison to a placebo [PLA] equivalent) would; (a) improve athletic performance, and (b) reduce post-exercise markers of inflammation, oxidative stress and perceptual muscle soreness.

## Methods

### Subject background and preparation

Nine highly-trained male Water Polo players were recruited from the Western Australian Institute of Sport (WAIS) Water Polo squad. The mean (± SD) physical characteristics of the participants were; age: 18.6 (±1.4) years, body mass: 82.7 (±9.8) kg and Σ_7_ skin-folds: 70.7 (±29.7) mm. All players were provided with an information sheet outlining the procedures, potential risks and benefits of the study prior to signing an informed consent agreement to participate. In the event that the participant was under the age of 18 years, signed consent was obtained from the individuals’ parent or legal guardian. Approval for the study was obtained from the Human Research Ethics Committee at the University of Western Australia (RA/4/1/7380).

### Experimental overview

A randomised double-blind, repeated measures, crossover design was employed to assess the influence of the tart CJ supplementation versus the PLA. The investigation consisted of two experimental trials, each involving a 7-day protocol. A 5-week washout period was adopted between the two experimental trials to preclude any follow-on effects, based on comparable previous supplementation regimes [6, 7]. During the experimental trials, participants were supplemented with CJ or a PLA equivalent from day 1 to day 6. On day 1, prior to the supplementation period, water-based performance testing was conducted. No form of physical activity was performed in the preceding 48 h of day 1, with participants arriving at the laboratory at 05:00 am to provide a venous blood sample immediately prior to commencing a standardised warm-up and the testing battery. The swimming-based tests comprised of the in-water vertical jump test (VJ), 10 m sprint test, the repeat sprint test (RST) and the Water Polo Intermittent Shuttle Test (WIST) [8], completed in the aforementioned order. All test protocols were conducted in accordance with the Australian National Water Polo Test Protocols [9, 10]. Capillary blood samples were collected to measure blood lactate levels (BLa) following the RST and WIST using a Lactate Pro II analyser (Arkray, Japan). All testing sessions were performed in a heated indoor Water Polo pool (27.5 °C). The testing battery was conducted in the morning (identical to typical training times) to minimise any diurnal influence on the test outcomes.

Throughout each experimental trial, participants had a fixed training regime (Table 1). All training (technical skill, weights, and swimming) performed during the 7-day trials was identical, and took place in the controlled environment of the indoor Water Polo pool or gymnasium. Training duration and a rating of perceived exertion (RPE) were collected after each training session to establish a quantified training load (RPE x Session Duration; [11]). The average daily training load was similar between trials (*p* > 0.05), at 561 ± 108 arbitrary units (AU) and 572 ± 89 AU for the CJ and PLA conditions, respectively. Participants also completed a comprehensive online daily diary for the duration of each experimental trial in order to assess their perceived recovery.

**Table 1:** Training schedule from day 1 to day 6 for both the cherry juice and placebo trial weeks

	Day 1	Day 2	Day 3	Day 4	Day 5	Day 6	Day 7
AM	Testing battery	Weights	Swim set	Weights	Training	Weights	Testing battery
	VJ	Sumo deadlift^A^	Kick set	Back squat^A^	Swim set (3.3 km)	Barbell step-up^A^	VJ
	10 m sprint	Bench press^A^	Freestyle	Push press dumbbell	Passing	Dumbbell bench press^A^	10 m sprint
	RST	Bench throw in smith machine^B^	Breaststroke	Push press medicine ball^D^	Game play	Bench pull: drop & catch^E^	RST
	WIST	Medicine ball chest pass	Backstroke	Pallof press^A^		Shoulder external rotation^F^	WIST
		Chin-ups^A^	(Total: 5 km, 90 min)	Single arm row^A^		Kneeling chop^G^	
		One arm kettlebell side bends^C^		Oblique twist with kettlebell^C^	(90 min)	Lateral pulldown^G^	
PM	Training	Training	Rest	Training	Rest	Match simulation	-
	Swim drills	Swim drills		Swim drills (1.8 km)		Warm-up	-
	Passing drills	Passing drills		Medicine ball holds		8 × 5 min quarters	-
	Leg strength	Leg strenght		Wrestling		Cool down	-
	Shooting	Shooting		Game play		(60 min)	-
	Game play	Game play		(90 min)			-
	(120 min)	(90 min)					-

*S* Sets, *R* Repititions, *RM* Repitition max, *BW* Body weight

(A) 4S × 4R at 75–80% 1RM

(B) 3S × 5R at ~50% BW

(C) 2S × 10R at 10–12 kg

(D) 3S × 5R at 10 kg

(E) 2S × 10R at 60% 1RM

(F) 3S × 10R at 4 kg

(G) 3S × 10R at ~30% BW

On day 6, participants attended the laboratory at 15:45 pm to provide a venous blood sample immediately prior to undertaking a simulated fatiguing team game activity, designed to replicate the demands of a Water Polo match [12]. A post-exercise venous blood sample was collected upon completion of the simulation. Finally, on day 7 of the supplementation period, participants arrived at the laboratory at 07:30 am to provide a final venous blood sample. Immediately thereafter, the athletes were required to re-perform the water-based performance tests as per day 1.

Venous blood samples collected throughout each experimental trial were used to investigate biological markers indicative of the efficacy of CJ. Inflammatory markers [high sensitivity (hs) IL-6 and CRP] were measured on day 6 (pre- & post-match simulation) and day 7 (12 h post-match simulation) of each testing week. Markers of oxidative stress [Uric acid (UA) and F_2_ isoprostane (F_2_-IsoP)] were measured at all 4 venous blood sampling time points; day 1 (pre-performance testing), day 6 (pre- & post-match simulation), and again on day 7 (pre-performance testing).

### Experimental procedures

#### Cherry juice supplementation

Participants consumed 90 mL daily of tart Montmorency CJ (Prunus Cerasus) concentrate (Cherry Active, Sunbury, UK) or a PLA equivalent for a total of 6 days. The cherry concentrate was diluted with water, such that each 30 mL serving was made up into a 200 mL beverage. Both the CJ and PLA were consumed in two doses each day; 200 mL before morning training, and 400 mL in the evening post-training. According to the manufacturer, a 30 mL dose of Cherry Active concentrate is equivalent to approximately 90 whole Montmorency tart cherries (Cherry Active, Sunbury, UK) containing 9.117 mg/mL of anthocyanins, which has previously been reported to have positive health and performance outcomes [4, 6, 13].

The PLA was made by combining 40 mL of three different ‘off the shelf’ cordials. Lime (Woolworths select lime cordial, Australia), cranberry (Bickford’s cranberry juice cordial, Australia) and raspberry (Cottee’s raspberry flavoured cordial, Australia) cordials were mixed with food colouring and 480 mL of water in order to closely imitate the taste, colour and carbohydrate content (10 g/100 mL, maltodextrin powder, Nutricia Poly-Joule, Australia) without any of the anthocyanin content of the CJ. Daily adherence to supplement consumption was made by the investigators being present at every session.

### Performance tests and match simulation

#### Testing battery

As previously highlighted, all four performance tests (VJ, 10 m sprint, RST, WIST) were specifically selected based on their ability to assess important traits of Water Polo [8–10]. A minimum of 5 min rest between each test was also adopted to ensure adequate recovery. The VJ test required participants to propel themselves as high out of the water as possible, extending their arms and fingers directly upward at the peak of the jump to displace as many vanes on a customised Water Polo specific Yardstick®; with the best attempt of three trials recorded. The 10 m sprint (best of two trials) required participants to swim between two ropes held just above water level at 0 and 10 m (that were raised/lowered by the research team). Similar in design to the 10 m sprint, the RST consisted of 6 × 10 m sprints departing every 17 s between the 0 and 10 m ropes. A digital video camera (Sony HDR-HC9, Japan) filming at 50 Hz was positioned on the opposite side of the pool, with the viewing width set to ensure both start and finish markers were clearly visible. Before testing, a calibration rope was also filmed allowing virtual lines to be accurately overlaid onto the video analysis program (Dartfish, Australia) at both 0 and 10 m for the accurate calculation of sprint time. Finally, WIST may be considered the water specific equivalent of the land-based Yo-Yo intermittent recovery test [9, 10]. The WIST lasts approximately 14 min in total (for this population) and consists of repeated 2 × 7.5 m shuttles (swims out and back) at progressively increasing speeds, interspaced by 10 s of recovery (treading water) that is controlled by audio signals. The protocols, validity and reliability of the water-based testing schedule have been previously established, and are currently adopted by Water Polo Australia [9]. Finally, specific details of the match simulation protocol have previously been reported [12]. The purpose of use here was solely to function as a fatiguing mechanism.

### Daily diaries

On waking, athletes provided a Total Quality of Recovery (TQR) rating and Delayed Onset Muscle Soreness (DOMS) score for the upper body, upper legs, lower legs and overall body. The TQR measured how well athletes felt they had recovered, and encompassed the anchor points 6 (very, very poor recovery) to 20 (very, very good recovery) [14]. The DOMS scale was included to measure how sore athletes felt, encompassing the anchor points of 0 (normal; without pain or stiffness) to 10 (very painful) [15].

### Blood analysis

Venous blood was collected from an antecubital vein using a 21-gauge needle into an 8 ml gel separator tube. All samples were subsequently centrifuged at 10 °C for 10 min at 3000 rpm, and stored in 1.5 mL eppendorfs at −80 °C until further analysis at a commercial pathology laboratory (PathWest Laboratory, Fiona Stanley Hospital). The IL-6 was analysed via immunoassay technique (Quantikine HS ELISA, R&D Systems, Inc. Minneapolis, USA). The coefficient of variation (CV) for inter-assay precision at 0.49 and 2.78 pg/mL was 9.6 and 7.2% respectively. The hsCRP was measured using an Architect analyser (ci8200), and determined using a CRP Vario Reagent (SENTINEL CH. SpA, Via Robert Koch, 2, Milan 20152, Italy). The CV for CRP determination at 0.88, 2.21 and 6.22 mg/L was 2.3, 1.2 and 1.0%, respectively. The UA was measured using an Architect analyser (ci8200), and determined using a UA Reagent (Abbott Diagnostics, Abbott Laboratories, Abbott Park, IL 60064, USA). The CV for UA determination at 0.25 and 0.56 mg/L was 1.92 and 1.5%, respectively. F_2_-IsoP was analysed using an Agilent 6890 gas chromatograph coupled to an Agilent 5973 mass selective detector. The mean total (free + esterified) plasma F_2_-IsoP concentration was 952 ± 38 pmol/L, with a within and between assay reproducibility of 8.0 and 5.6%, respectively [16].

### Statistical analysis

Results are expressed as mean (±SD) and were analysed using a repeated measures analysis of variance (ANOVA) to determine time, condition and interaction effects of tart CJ on measures of recovery and performance. A post-hoc paired samples *t*-test was used to determine any differences between trials. The alpha level was accepted at *p* < 0.05.

## Results

### Blood variables

**Fig. 1:**
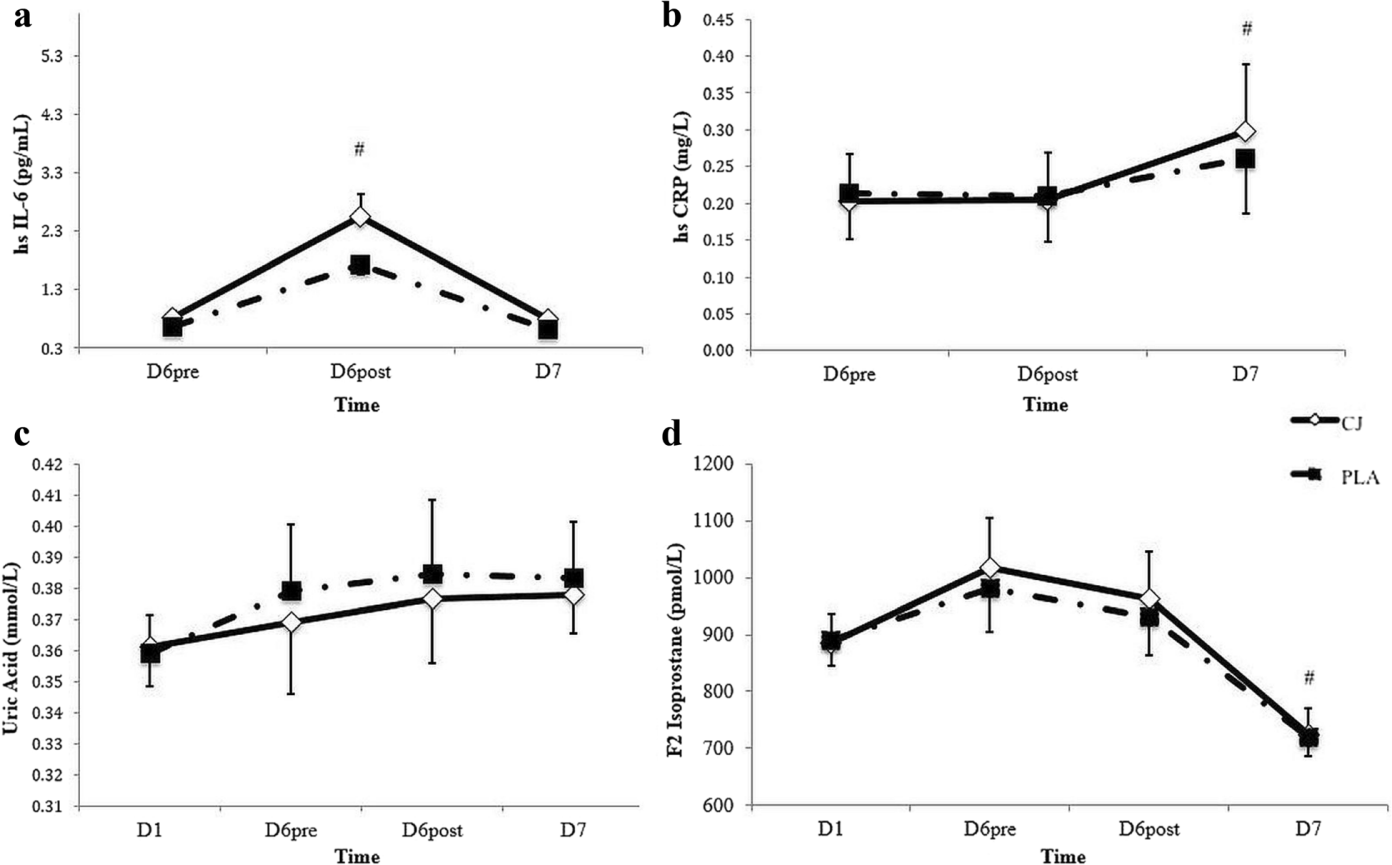
Levels of high sensitivity (**a**) Interleukin-6 (**b**) C-Reactive protein (**c**) Uric acid and (**d**) F_2_-Isoprostane on day 1 (D1), day 6 pre-exercise (D6pre), day 6 post-exercise (D6post). #Significantly different to (**a**) D6pre and D7 (**b**) D6pre and D6post; and (**d**) D6pre and D6post, time-points in both the CJ and PLA trials

The levels of IL-6, CRP, UA and F_2_-IsoP are depicted in Fig. 1 (a), (b), (c) and (d), respectively. For IL-6, CRP and F_2_-IsoP, a significant time (*p* < 0.05), but no condition or interaction effect, was apparent across the supplementation period. Specifically, IL-6 levels were significantly greater (*p* < 0.05) on day 6 post-exercise as compared to day 6 pre-exercise and day 7. Additionally, CRP levels were significantly greater on day 7 as compared to those measured on day 6 pre- and post-exercise (*p* < 0.05). Furthermore, F_2_-IsoP levels were significantly lower (*p* < 0.05) on day 7 as compared to those recorded on day 1 and day 6 pre- and post-exercise. Finally, no condition, time or interaction effects were evident for UA.

### Performance variables

Performance data for the numerous Water Polo tests are shown in Table 2. No condition, time or interaction effects were found for the WIST, VJ, 10 m sprint and RST scores achieved across the supplementation period. However, analysis of post-WIST and RST BLa levels showed a significant effect for time (*p* < 0.05) between day 1 and day 7; such that BLa levels were significantly lower on day 7 (*p* < 0.05) compared to day 1 across both conditions.

**Table 2:** Mean (±SD) performance for Vertical Jump (VJ), Water Polo Intermittent Swim Test (WIST), Repeat Swim Test (RST) and 10 m sprint for the cherry juice and placebo condition on day 1 and day 7

Test variable		Cherry juice		Placebo	
		Day 1	Day 7	Day 1	Day 7
VJ	Absolute (cm)	149 ± 7	150 ± 6	149 ± 6	150 ± 6
	Decimal	10.0 ± 2.2	9.5 ± 2.0	9.9 ± 1.2	9.2 ± 1.7
WIST	Distance (m)	655 ± 261	605 ± 239	643 ± 151	558 ± 203
	BLa (mmol/L)	6.3 ± 1.8	4.2 ± 1.5^a^	5.2 ± 1.7	3.7 ± 1.4^a^
RST	Total time (s)	37.09 ± 1.92	36.76 ± 0.75	36.90 ± 0.78	37.07 ± 1.16
	Deficit time (s)	1.54 ± 0.70	1.50 ± 0.44	1.52 ± 0.61	1.56 ± 0.48
	Decrement (%)	4.34 ± 1.97	4.25 ± 1.29	4.31 ± 1.81	4.58 ± 1.50
	BLa (mmol/L)	4.2 ± 1.7	3.4 ± 0.7^a^	3.6 ± 0.6	3.3 ± 0.8^a^
10 m Sprint	Total time (s)	5.56 ± 0.15	5.59 ± 0.22	5.48 ± 0.24	5.56 ± 0.15

^a^Significantly different to Day 1 of each trial

### Perceptual variables

Ratings of DOMS and TQR are displayed in Table 3. No condition, time or interaction effects (*p* > 0.05) were found for the respective ratings for the duration of CJ or PLA supplementation.

**Table 3:** Mean (±SD) perceived ratings of Total Quality of Recovery (TQR) and Delayed Onset Muscle Soreness (DOMS) for the cherry juice and placebo condition on day 1 to day 6

	Cherry juice		Placebo	
	TQR	DOMS	TQR	DOMS
Day 1	16 ± 3	3 ± 2	15 ± 3	3 ± 2
Day 2	15 ± 4	3 ± 2	15 ± 3	4 ± 2
Day 3	15 ± 3	3 ± 2	17 ± 2	3 ± 2
Day 4	15 ± 3	3 ± 2	13 ± 4	4 ± 2
Day 5	15 ± 3	3 ± 2	15 ± 3	3 ± 2
Day 6	16 ± 4	3 ± 2	15 ± 3	4 ± 2

## Discussion

The findings of this study show that 6 consecutive days of tart CJ supplementation has no effect on athletic performance or recovery in highly-trained Water Polo athletes. This outcome is in contrast to previous research, and in part, may be related to different dosage strategies, and the modality and duration of exercise used here.

Previous research [2, 4] has shown CJ supplementation to be beneficial to athletic recovery in various sporting activities, reporting lower levels of the inflammatory marker IL-6 in the post-exercise recovery period when CJ was consumed. Specifically, Howatson et al. [2] found post-exercise IL-6 levels to be ~50% lower in marathon runners supplemented with CJ (2 × 240 ml serve of CJ daily over 8 days; one serve contained ~600 mg of phenolic compounds and ~40 mg anthocyanins) when compared to a PLA equivalent. Further, Bell, Walshe et al. [4] demonstrated that post-exercise IL-6 concentration was 200% lower in cyclists that were supplemented with CJ (2 × 30 ml of Cherry Active concentrate for 7 days) as compared to PLA, following 3 consecutive days of a simulated cycling road race (daily duration of 101 min comprising 66 sprints of various lengths ranging from 5 to 15 s, 2 × 4 min and 1 × 5 min time-trials). Both studies also reported reduced oxidative damage following exercise when CJ was consumed; an outcome supported by similar findings following maximal eccentric exercise [6].

To our knowledge, this is the first study examining the effects of CJ supplementation on inflammation, oxidative stress and next-day performance in team-sport athletes. Contrary to our hypothesis (and previous research), this study was unable to show a post-exercise attenuation in circulating levels of IL-6 and CRP, nor any influence on post-exercise oxidative stress with supplementary CJ. Such findings may be linked to the magnitude of inflammation, oxidative stress and muscle damage induced here, potentially being substantially lower when compared to previous studies. Possibly, the mechanical strain induced by Water Polo activity is likely to have been lower when compared to endurance running [2] or cycling [4] efforts, as a result of the weight-supported and intermittent nature of the activity performed. In support of this notion, Nieman et al. [17] suggested that the mechanical trauma incurred during exercise significantly modulates the magnitude of the inflammatory response; possibly explaining why post-exercise IL-6 levels have been reported ~40 times higher [2], and CRP levels reported ~6 times higher [4], than those reported after the simulated Water Polo match completed here. Contrary to our results, these studies suggest that any activity resulting in high levels of inflammation or muscle damage, such as marathon running (where IL-6 and CRP were ~50 and 35% lower with CJ, respectively [2]) or endurance cycling (where IL-6 and CRP ~50 and 80% lower with CJ, respectively [4]), would still benefit from CJ supplementation. However, the smaller degree of inflammation induced in our participants could potentially have been inadequate to have benefitted from any anti-inflammatory effects provided by the CJ supplementation. Additionally, the activity profile of Water Polo is significantly different to endurance exercise, consisting of a more intermittent activity pattern where players commonly perform ~100 sprint efforts of 7–14 s, interspersed by lower intensity activity over the duration of a match [18]. When related to markers of oxidative stress, the only somewhat comparable previous study adopted a high-intensity, intermittent cycling protocol (three sets of 9 × 5 s departing every 25 s) and observed a similar response for F_2_-IsoP [19]. Nevertheless, our results highlight the possibility that any associated benefits of CJ on performance/recovery may only be present in sports where substantially higher levels of inflammation and muscle damage might occur. To this end, future research should examine the efficacy of CJ supplementation on athletic performance and recovery in running-based (weight-bearing) team sports such as football, netball, hockey or rugby.

Reduced inflammation and oxidative stress post-exercise allows for the maintenance of muscular function and likely mitigates soreness, possibly explaining the preservation and recovery of strength found in previous studies where CJ was consumed [7]. That said, the lack of difference between the CJ and PLA groups on ratings of DOMS and TQR in the current study are likely due to the lack of inflammation and oxidative stress induced. These results are in contrast to the findings of Connolly et al. [7], who found that when participants performed repeated maximal eccentric contractions of their elbow flexors, the development of soreness (rated on a scale of 1 to 10) was significantly diminished in the group supplemented with commercially available CJ (~350 ml daily of Cherrypharm for a total of 8 days) as compared with the PLA. These authors also found that maximal isometric strength loss was attenuated in the 96 h following the CJ supplementation (4% vs. 22% decrement for the CJ vs. PLA supplement, respectively). Of interest, our results concur with Howatson et al. [2], who found no significant differences in muscle soreness in marathon runners that were supplemented with either CJ or a PLA. However, due to the aforementioned differences in exercise modalities, duration, muscle damage and inflammatory response between activities, it is difficult to make further definitive assumptions.

Another potential explanation for our results may be the dosage protocol employed. Anti-inflammatory and antioxidant effects have been found in marathon runners supplemented for 5 days before and 2 days after a race [2], and in cyclists supplemented for 4 days before and on each of 3 subsequent time-trial days [4]. Unlike these studies that continued to supplement during the performance and recovery periods, our participants consumed their final CJ dose in the evening following the Water Polo match simulation (day 6) prior to the performance testing (morning of day 7), as we wanted to specifically investigate next-day performance. Subsequently, this may have influenced the recovery outcomes, since the levels of CRP often continue to rise to a peak at 24 h post-exercise [2, 4]. However, it is suggested that the supplementation duration (6 days) and dosage (90 ml of CJ concentrate daily) used here would have been sufficient to show any potential benefits. Our dosage was equivalent to 270 cherries and 820 mg of anthocyanins daily (accounting for Water Polo players being heavier than endurance athletes) as opposed to previous investigations where performance benefits were recorded equating to a lower dose of 120 cherries and 80 mg of anthocyanins daily [2].

A limitation of this investigation is that the anthocyanin concentration of both the commercial and placebo supplement used was not confirmed. However, the concentration of the active ingredient in the tart cherry juice supplement used here has been previously published, and was therefore used due to the positive results from this research [4, 12]. Additionally, the placebo supplement was selected because the manufacturer of these cordials suggest there was no known anthocyanin content in their products. Furthermore, no blood analysis of supplement efficacy on increasing plasma anthocyanin levels was possible here; however, adherence to supplement consumption was confirmed by direct investigator contact with athletes at every training session. Regardless, future research must further consider these points moving forward.

## Conclusion

In summary, CJ supplementation had no significant effect on the recovery of Water Polo specific athletic performance. Unlike previous research, the current study showed no difference in anti-inflammatory or antioxidant activity in athletes supplemented with CJ compared with the PLA, thereby precluding any potential benefits to performance or recovery in Water Polo players. As such, our results suggest that CJ supplementation may not be necessary for water-based non-weight bearing intermittent sports such as Water Polo. Regardless, future research should examine the use of CJ in other running-based weight-bearing team sports before CJ can be recommended or excluded as an effective mechanism to improve recovery and next-day team sport performance.

## Abbreviations

AU: Arbitrary units
BLa: Blood lactate
CJ: Cherry juice
CRP: C reactive protein
DOMS: Delayed onset muscle soreness
F_2_-Isop: F_2_-isoprostane
hs IL6: High sensitive Interleukin 6
PLA: Placebo
RPE: Rate of perceived exertion
RST: Repeat sprint test
TQR: Total quality of recovery
UA: Uric acid
VJ: Vertical jump
WIST: Water Polo intermittent shuttle test
